# Evolutionary Game Analysis of Carbon Emission Reduction between Government and Enterprises under Carbon Quota Trading Policy

**DOI:** 10.3390/ijerph19148565

**Published:** 2022-07-13

**Authors:** Na Yu, Jianghua Chen, Lei Cheng

**Affiliations:** College of Economics and Management, Hefei University, Hefei 230601, China; cl394744@163.com

**Keywords:** evolutionary game, carbon trading, carbon emission reduction, simulation

## Abstract

As one of the most efficient means of emission reduction policies, carbon quota trading has a far-reaching impact on the carbon emission reduction of enterprises. Firstly, a two-party evolutionary game model of enterprise and government and a three-party evolutionary game model of enterprise–enterprise–government are constructed based on the multi-agent driving mechanism, evolutionary game theory, scenario simulation, and other methods. Then, we conduct a series of policy simulations for carbon emission under different scenario models and various enforcement strengths. Lastly, the behavioral strategies and system evolution trajectories in enterprises and government carbon trading are comprehensively investigated. The results show that in the two-party and three-party evolutionary game models, the carbon trading behavior is affected by the joint action of the enterprise and the government. The difference in initial willingness mainly affects the speed of the subject’s convergence to the steady state. Based on this, policy suggestions are proposed, such as reducing the cost of carbon emission of enterprises, enhancing the vitality of carbon emission reduction of enterprises, and stimulating the power of government regulation and responsibility performance, which can provide suggestions for the development of the carbon market.

## 1. Introduction

With the continuous development and progress of the economy, the environmental problems caused by carbon dioxide emissions have received more and more attention and discussion [1,2,3,4,5]. In September 2020, China clearly put forward the dual-carbon goal, striving to reach the peak of carbon dioxide emissions by 2030 [6] and strive to achieve carbon neutrality by 2060 [7,8,9]. During the 14th Five-Year Plan, the goal of “reducing energy consumption per unit of GDP by 13.5% and carbon dioxide emissions by 18%” was set. In this regard, the establishment of a carbon emission rights trading market (referred to as the carbon market) is a key tool to effectively solve the problem of carbon dioxide emissions [10,11,12]. In July 2021, the carbon market officially launched online trading, which means that the national unified carbon market trading system is gradually formed. From the perspective of transaction volume and transaction value, as of 31 December 2021, the national carbon market has been running for 114 trading days, the cumulative transaction volume of carbon emission allowances is 179 million tons, and the cumulative transaction value is 7.661 billion yuan. Hence, it is of theoretical importance and practical value to explore the use of the carbon trading market mechanism to achieve green and efficient development under the dual pressures of economic development and environmental protection [13,14,15].

In recent years, carbon emission reduction has become one of the hot topics discussed by scholars, and preliminary progress has been made in the research on carbon emission reduction mechanisms and methods. For example, Barragán et al. constructed a comparative assessment of carbon taxes and emissions trading schemes from an economic and political perspective, and the results showed that the emissions trading scheme was the most suitable tool for the Mexican case. Compared to carbon taxes, the emissions trading scheme had higher cost-efficiency and lower distributional effects [16]. Shahbaz et al. analyzed the impact of financial inclusion on the synergistic emission reduction of pollutants and carbon emissions, using data from 30 provinces in China from 2011 to 2017, and concluded that financial inclusion achieved the collaborative reduction of pollutants and carbon emissions reductions [17]. Li pointed out that local government decision-making competition was the key factor in carbon emissions in the new urbanization process and proposed that the impact mechanism of local government decision-making competition on carbon emissions through a nonlinear programming model. The results showed that local government decision-making competition affected carbon emissions by distorting factor markets, government investment, and environmental policy implementation [18]. Gu et al. examined the energy saving and emission reduction effects of the carbon financial market as an environmental regulatory policy tool, and the results revealed that carbon market policies continued to have a positive impact on energy conservation and emission reduction. After the implementation of the carbon policy, each pilot area produced different degrees of energy saving and emission reduction effects [19]. Wang and Hui constructed a manufacturer–retailer supply chain system considering carbon footprint, aiming to study the impact of joint emission reduction on the emission reduction level and income of retailers and manufacturers. The results illustrated that the higher the manufacturer’s emission reduction level, the smaller the carbon footprint, thereby improving the low carbon level, increasing the sales scale, and thus enhancing the manufacturer’s emission reduction enthusiasm [20]. Zhu et al. focused on the multiple impacts of urbanization on carbon emissions, using spatial panel data combined with remote sensing data of nighttime lights in Zhejiang Province from 1995 to 2015. The results showed that urban population growth and low carbon awareness could reduce carbon emissions [21]. Li and Wang found that carbon trading policies promoted carbon emissions in pilot areas by studying carbon trading pilot data, and technological progress was the key to implementing the effect of carbon emission reduction [22]. Zhang et al. discussed the impact of the digital economy on carbon emissions performance and found digital economy improved carbon performance mainly through energy intensity, the scale of energy consumption, and urban afforestation; the digital economy had a significant effect on the carbon emissions performance of local cities, but not in neighboring cities [23]. Liu et al. studied the impact of changes in China’s sustainable economic development goals on the energy conservation and emission reduction behavior of Chinese enterprises, mainly by optimizing the consumption energy structure and increasing investment in green technology [24].

For the research on carbon emission reduction methods, Liu et al. used the Stackelberg game model to construct an analytical framework composed of manufacturers and retailers, and the optimal emission reduction mode selection under the carbon emission cap and trade mechanism was discussed; that is, the joint emission reduction level is the highest [25]. Linghu et al. established a mixed game model and analyzed the impact of firm heterogeneity and social willingness to pay for carbon trading mechanisms. The results found that reasonable allocation of carbon allowances is an effective tool to motivate industry and consumers [26]. Li and Wang established the spatial DURBIN model and panel threshold model to analyze the impact of the digital economy on carbon reduction. The empirical results showed that the digital economy had an inverted U-shaped relationship with carbon emissions, and the spatial spillover effect of the digital economy on carbon emissions was also inverted U-shaped [27]. Yang et al. explored the agricultural carbon emissions by DEA–Malmquist–Luenberger and panel data analyses, and the results showed that the agricultural carbon emission intensity began to show a turning point in 2015, showing a downward trend [28]. Wang et al. constructed the framework of the Stackelberg model, taking into account the constraints of carbon allowances, and designed the carbon emission coordination game mechanism for coal-fired power supply chain enterprises [29]. Jin et al. presented a distributed robust optimization model to discuss the allocation of initial carbon emission rights; the analysis showed that carbon trading was an effective way to reduce carbon emissions [30]. Wang et al. studied the carbon emission reduction of the building supply chain with the help of differential game theory and MATLAB numerical simulation research, factors such as centralized decision making, decentralized decision making, and cost-spreading contracts were introduced into the differential game model, and the aim is to find the best trajectory for carbon reductions over time [31]. Luo et al. used a difference game to analyze carbon emission reduction decisions of supply chain members under the cap-and-trade system. It was found that, compared with non-cooperation, two enterprises that choose to cooperate could make more emission reduction efforts and gain more profits [32]. Song et al. constructed an evolutionary game model, analyzed the synergistic effects of regulatory policies, and used system dynamics to conduct a simulation analysis. The results showed that interventions such as punishment, subsidies, and public supervision were more effective for the government [33].

By reviewing the literature, it is found that scholars have carried out relevant research on carbon trading mechanisms and carbon emission reduction methods. For carbon emission reduction, the Stackelberg game model, EDA model, and panel data model are mainly used. However, few scholars have introduced evolutionary game theory into carbon trading. More importantly, there is a lack of research on the mechanism of interaction between government and enterprises in carbon trading. In fact, sorting out the game relationship between government and enterprises in carbon trading not only helps to explore the optimal path for carbon emission reduction but also provides ideas for the development of the carbon market [34]. Therefore, the present study incorporates government behavior into the unified analysis framework of carbon trading, constructs two-party and three-party evolutionary game models of enterprise–government and enterprise–enterprise–government, and explores the practical problems faced in the game of carbon emission reduction between government and enterprise.

The innovations of the present study mainly include the following three aspects: Firstly, it is assumed that two enterprises are homogeneous in the evolutionary game model and have the same transaction status and transaction authority. Secondly, the study establishes enterprise–government and enterprise–enterprise–government tripartite evolutionary game models and explores the evolution paths of enterprises and governments from a multi-agent perspective. Finally, the evolution paths of enterprises and governments are visualized by using the three-dimensional graphic method, and the potential mechanisms of carbon emission are analyzed deeply. The coping strategies for evolution scenarios are proposed to provide policy suggestions for better promoting carbon trading and enhancing new momentum for green development.

## 2. Multi-Agent Driving Mechanism of Carbon Trading

This paper elucidates the multi-agent driving mechanism of carbon trading from two perspectives: the evolution mechanism of government participation driving enterprise carbon trading and the evolution mechanism of enterprise main body driving carbon trading, exploring the multi-agent interaction relationship between enterprise–government and enterprise–enterprise–government, and constructing the theoretical analysis of this paper The framework provides a theoretical basis for the construction of two-party and three-party evolutionary game models of carbon trading below.

### 2.1. Government Participation Drives the Evolution Mechanism of Carbon Trading

Carbon emission is a negative externality behavior of production [35]; that is, the marginal social cost of carbon emission is greater than the marginal private cost, and the carbon emission behavior of enterprises affects other people or enterprises but does not bear the corresponding costs for it, which will lead to carbon emission [36,37]. Excessive supply leads to unreasonable resource allocation and loss of efficiency [38]. According to the government’s paternalistic view, enterprises will often be unwise, have no foresight, lack sufficient motivation to implement carbon emission reduction, and will not take the initiative to conduct carbon emission reduction transactions. The government’s participation has solved the problem of negative externalities of the environment. For example, by formulating a series of incentive policies to support enterprises’ carbon emission reduction trading, appropriate subsidy is an effective means to encourage enterprises to implement carbon emission reduction trading and ultimately achieve carbon emission reduction [39,40].

In December 2014, the National Development and Reform Commission of China organized the drafting and implementation of the Interim Measures for the Administration of Carbon Emissions Trading [41]. In February 2021, the “Measures for the Administration of Carbon Emissions Trading (Trial)” were implemented, which stipulates that the trading products in the national carbon emission trading (referred to as carbon trading) market are carbon emission allowances, which are mainly distributed free of charge [42], and paid distribution is introduced in due course [43]. The quota trading system has been adopted by many countries [44], such as the EU’s implementation of the Greenhouse Gas Emissions Trading Scheme (i.e., EU-ETS) [45], which is used to motivate and urge enterprises to reduce carbon emissions [7,46].

### 2.2. Evolution Mechanism of Enterprise Entities Driving Carbon Trading

The carbon trading mechanism is an environmental regulation in the form of a market [47], which will promote the internalization of carbon emission reduction costs [48]. Enterprises with high emission reduction costs complete emission reduction tasks by purchasing carbon allowances, and enterprises with low emission reduction costs undertake more emission reduction tasks and gain profits. The enterprises have to bear the full cost of carbon emissions to society, which helps to realize the rational allocation of resources and ultimately achieve the purpose of reducing the intensity of carbon emissions.

In the development process of the carbon market, positive and negative incentives such as carbon trading policies [49], financial subsidy policies [50], and punishment policies are coordinated to supervise the disclosure of carbon information and directly affect business behavior through compliance costs [51]. As the main body of carbon trading implementation, the enterprises will make decisions based on their own interests. The carbon trade fair will force enterprises to change the traditional form of extensive energy emissions, promote enterprises to eliminate outdated processes, increase investment in environmental protection technologies, innovate low-carbon technologies, develop low-carbon products and low-carbon services, reduce carbon emissions, and reduce the remaining carbon emissions. The emission credits translate into economic benefits. Low-carbon enterprises can gain a competitive advantage in the market, be recognized by the society, produce technological leadership and demonstration effects, drive more enterprises to carry out technological innovation, and produce a diffusion effect of carbon emission reduction [52].

## 3. Construction and Analysis of Evolutionary Game Model

It is seriously out of reality to have a completely rational assumption to analyze the final stable state of the subject due to the complex environment faced by enterprises and government actors [53,54]. The traditional game theory based on the assumption of complete rationality has poor reliability [55]. However, the evolutionary game theory based on the assumption of bounded rationality can effectively solve the problem of stable decision making. The evolutionary game is from the perspective of biological evolution theory [56], through continuous learning and evolution among individuals [57], and finally reaches a stable state and forms an evolutionarily stable strategy [58,59,60]. Therefore, based on the multi-agent evolution mechanism of carbon trading, this paper sets up the enterprise–government two-party evolutionary game model and the enterprise–enterprise–government three-party evolutionary game model to explore the multi-agent game equilibrium of carbon trading behavior [61].

### 3.1. Parameter Description

The parameters involved in the government and enterprises are explained, and the relevant parameter symbols and definitions are shown in Table 1.

### 3.2. Basic Assumptions of the Model

**Hypothesis** **1** **(H1).** *Participating subjects. There are three types of participants in the carbon trading process, namely government environmental regulation departments, carbon emission enterprise 1, and carbon emission enterprise 2. The government, as the supervision department of carbon trading behavior* [62]*, chooses to implement a supervision or non-supervision strategy* [63]*. Enterprises, as the main body of carbon emission, choose whether to implement carbon emission reduction or not. The government, enterprise 1, and enterprise 2, as the subject of limited knowledge and bounded rational behavior, have the problem of information asymmetry and constantly adjust their own strategies according to the behavior of the other party in the game process. Therefore, it is assumed that the government, enterprise 1, and enterprise 2 conduct game analysis based on bounded rationality.*

**Hypothesis** **2** **(H2).** *Cooperative strategy. During the implementation of carbon reduction, enterprise 1 and enterprise 2 can choose to reduce carbon emissions based on limited knowledge, or they can choose not to reduce carbon emissions and choose to purchase carbon indicators. The enterprise strategy set is (reduction and purchase). The government environmental regulation department can choose to regulate the carbon emission reduction of enterprises based on limited information and can also choose not to regulate the carbon emission reduction of enterprises. The set of government strategies is (regulation and non-regulation), in which regulation means that the government implements subsidy incentives for positive carbon emission reduction enterprises and takes punitive measures for negative emission reduction enterprises.*

**Hypothesis** **3** **(H3).** *Project benefits. Assuming that the enterprise chooses not to reduce emissions, the comprehensive revenue of the enterprise is*$E_{0}^{F}$*, and the revenue of the government is*$E_{0}^{G}$*. If enterprises choose to actively reduce emissions, the ecological revenue and product quality of the enterprises will be improved accordingly. The increase in revenue for the enterprise is*$\Delta E_{0}^{F}$*, and the increase in revenue for the government is*$\Delta E_{0}^{G}$*. If some enterprises choose to actively reduce emissions, and some enterprises choose not to reduce emissions but to purchase carbon quotas, then the increased revenue of the government is*$\Delta E_{1}^{G}$*, and the increased revenue of emission reduction enterprises is*$\Delta E_{1}^{F}$*. At the same time, if the government chooses to actively subsidize the emission reduction enterprises* [64]*, the subsidy is recorded as* S*. If the government chooses to take punitive measures against the emission reduction enterprises, the penalty is recorded as* F*.*

**Hypothesis** **4** **(H4).** *Project cost. If the government implements active regulation on emission reduction enterprises, it will inevitably pay human and material resources and other costs* [62]*, denoted as* $C_{1}^{G}$*. If the government does not implement regulations on whether enterprises reduce emissions, the reputation cost and economic cost of the government’s loss are denoted as* $C_{2}^{G}$*. In addition, if the enterprise implements active emission reduction, it will pay the corresponding cost, denoted as* $C_{1}^{F}$*. If the enterprise does not reduce emissions, the enterprise will have a negative evaluation, and the economic loss brought by it is denoted as* $C_{2}^{F}$.

**Hypothesis** **5** **(H5).** *Transaction mechanism. Carbon trading refers to the sale of carbon allowances by enterprises with sufficient carbon allowances to enterprises with limited carbon allowances* [65,66]*. Assuming that the carbon quota allocated by the government to the enterprise for free is* $Q_{0}$*, the carbon emission when the enterprise actively reduces emissions is* $Q_{1}$*, and the carbon emission when the enterprise does not reduce emissions is* $Q_{2}$*, which is* $Q_{1}<Q_{0}<Q_{2}$*. At the same time, assuming the carbon trading price, if the government implements regulatory policies on carbon emission reduction enterprises, the government can obtain profits from carbon trading, which is the profit distribution coefficient. At the same time, assuming that* P *is the carbon trading price* [67]*, if the government implements regulatory policies on carbon emission reduction enterprises, the government can obtain profits from carbon trading, and* α *is the profit distribution coefficient* [68].

### 3.3. Evolutionary Game Model between Government and Enterprises

In the evolutionary game model between the government and the enterprise, it is assumed that the carbon-emitting enterprise chooses to reduce carbon emissions with the probability of x∈[0,1], and chooses not to reduce carbon emissions but purchase carbon indicators with the probability of (1−x). The government regulates carbon emission reduction enterprises with the probability of y∈[0,1], and chooses not to regulate with the probability of (1−y). According to the above assumptions, the evolutionary game payoff matrix between the government and enterprises is obtained [69], as shown in Table 2.

The expected and average revenue of enterprise emission reduction and carbon purchases are as follows:

(1) $$\begin{array}{ll} U_{F1}= & y[E_{0}^{F}+\Delta E_{0}^{F}-C_{1}^{F}+S+P(Q_{0}-Q_{1})]\\ & +(1-y)(E_{0}^{F}+\Delta E_{0}^{F}-C_{1}^{F}) \end{array}$$

(2) $$\begin{array}{ll} U_{F2}= & y[E_{0}^{F}-C_{2}^{F}-F-P(Q_{2}-Q_{0})]\\ & +(1-y)(E_{0}^{F}-C_{2}^{F}) \end{array}$$

(3) $${\bar{U}}_{F}=xU_{F1}+(1-x)U_{F2}$$

The expected and average revenue of government regulation and non-regulation are as follows:

(4) $$\begin{array}{ll} U_{G1}= & x[E_{0}^{G}+\Delta E_{0}^{G}-C_{1}^{G}-S+\alpha P(Q_{0}-Q_{1})]\\ & +(1-x)[E_{0}^{G}-C_{1}^{G}+F+\alpha P(Q_{2}-Q_{0})] \end{array}$$

(5) $$\begin{array}{ll} U_{G2}= & x(E_{0}^{G}+\Delta E_{0}^{G}-C_{2}^{G})\\ & +(1-x)(E_{0}^{G}-C_{2}^{G}) \end{array}$$

(6) $${\bar{U}}_{G}=yU_{G1}+(1-y)U_{G2}$$

The dynamic evolutionary game between the enterprise and the government can be described by means of the replication dynamic equation [70]. The replication dynamic equation of the enterprise is expressed as:

(7) $$\begin{array}{ll} F(x) & =dx/dt\\ & =x(1-x)[y(S+F+PQ_{2}-PQ_{1})+\Delta E_{0}^{F}+C_{2}^{F}-C_{1}^{F}] \end{array}$$

The government’s replication dynamic equation is expressed as:

(8) $$\begin{array}{ll} F(y) & =dy/dt\\ & =y(1-y)[x(2\alpha PQ_{0}-\alpha PQ_{1}-\alpha PQ_{2}-S-F)+\alpha PQ_{2}+F-\alpha PQ_{0}-C_{1}^{G}] \end{array}$$

The game between the enterprise and the government can be represented by a differential equation composed of F(x), F(y), and F(z). Through the local stability of the Jacobian matrix, the stable strategy of the system evolution is obtained, which is expressed as:

$$J=\left\{\begin{array}{cc} \begin{array}{l} (1-2x)[y(S+F+PQ_{2}\\ -PQ_{1})+\Delta E_{0}^{F}+C_{2}^{F}-C_{1}^{F}] \end{array} & \begin{array}{l} x(1-x)(S+F+PQ_{2}-PQ_{1}) \end{array}\\ \begin{array}{l} y(1-y)(2\alpha PQ_{0}-\alpha PQ_{1}\\ -\alpha PQ_{2}-S-F) \end{array} & \begin{array}{l} (1-2y)[x(2\alpha PQ_{0}-\alpha PQ_{1}\\ -\alpha PQ_{2}-S-F)+\alpha PQ_{2}+\\ F-\alpha PQ_{0}-C_{1}^{G}] \end{array} \end{array}\right\}$$

Set the replication dynamic equation of enterprise and government to zero [71]; four partial equilibrium solutions can be obtained, namely $E_{1}(0,0)$, $E_{2}(0,1)$, $E_{3}(1,0)$, and $E_{4}(1,1)$. The eigenvalues of the Jacobian matrix obtained from the equilibrium points are shown in Table 3.

**Type** **1.** $\Delta E_{0}^{F}<C_{1}^{F}-C_{2}^{F}<\Delta E_{0}^{F}+S+F$, $\alpha P(Q_{2}-Q_{0})+F<C_{1}^{G}<\alpha P(Q_{0}-Q_{1})-S$.

When the difference between the cost of emission reduction and no emission reduction is between the increased revenue when the enterprise chooses emission reduction and the sum of the increased revenue when the enterprise chooses emission reduction, the government’s subsidies to emission reduction enterprises, and the government’s punishment for non-emission reduction enterprises when the regulatory cost of the government’s supervision of enterprise emission reductions is between the sum of the government’s carbon trading revenue and the government’s punishment for enterprises that do not reduce emissions and the difference between the government’s carbon trading revenue and the government’s subsidies for emission reduction enterprises, there are two evolutionary stabilization strategies for the enterprise and the government, respectively, $E_{1}(0,0)$ and $E_{4}(1,1)$, as shown in Table 4. That is, the enterprise chooses not to reduce emissions, and the government chooses not to regulate; or the enterprise chooses to reduce emissions, and the government chooses regulation.

**Type** **2.** $C_{1}^{F}-C_{2}^{F}>\Delta E_{0}^{F}+S+F+P(Q_{2}-Q_{1})$, $F+\alpha P(Q_{2}-Q_{0})>C_{1}^{G}$.

When the difference between the cost of implementing emission reduction and non-emission reduction is higher than the sum of the increase in revenue when the enterprise chooses to reduce emission, the government’s subsidies to emission reduction enterprises, the government’s punishment for non-abatement enterprises, and the benefits of carbon trading, moreover, when the sum of the government’s punishment for non-emission reduction enterprises and the government’s income from carbon trading is higher than the government’s regulation cost for emission reduction enterprises, the stable strategy of the evolutionary game between the enterprise and the government is $E_{2}(0,1)$. That is, the enterprise does not choose to reduce emissions, and the government chooses regulation.

**Type** **3.** $C_{1}^{F}-C_{2}^{F}<\Delta E_{0}^{F}$, $\alpha P(Q_{0}-Q_{1})<S+C_{1}^{G}$.

When the difference between the cost of implementing emission reduction and non-emission reduction is lower than the increased revenue when the enterprise chooses to reduce emissions, when the government’s income from carbon emission reduction is lower than the sum of the government’s subsidies for emission reduction enterprises and the government’s regulation cost to emission reduction enterprises, the stable strategy of the evolutionary game between the enterprise and the government is $E_{3}(1,0)$. That is, the enterprise chooses to reduce emissions, and the government chooses not to regulate.

### 3.4. The Tripartite Evolutionary Game Model between the Government and Both Enterprises

In the evolutionary game model, carbon-emitting enterprise 1, carbon-emitting enterprise 2, and the government make strategic choices according to their own wishes. It is assumed that carbon-emitting enterprise 1 chooses to reduce carbon emissions with the probability of x∈[0,1], and chooses not to reduce carbon emissions with the probability of (1−x). Enterprise 2 chooses to reduce carbon emissions with the probability of y∈[0,1], and chooses not to reduce carbon emissions with the probability of (1−y). The government regulates carbon emission reduction enterprises with the probability of z∈[0,1] and chooses not to regulate with the probability of (1−z). According to the above assumptions, the evolutionary game payment matrix of carbon trading among enterprises is obtained [72], as shown in Table 5 and Table 6.

According to the evolutionary game payment matrix of carbon trading between enterprises, the expected return of the government choosing regulation, the expected return of choosing non-regulation, and the average expected return of the government in the evolutionary game are calculated as:

(9) $$\begin{array}{l} E_{G1}=xy[E_{0}^{G}+\Delta E_{0}^{G}-C_{1}^{G}-S+\alpha P(Q_{0}-Q_{1})]+x(1-y)\\ \left[E_{0}^{G}+\Delta E_{1}^{G}-C_{1}^{G}-S+F+\alpha P(Q_{0}-Q_{1})+\alpha P(Q_{2}-Q_{0})\right]\\ +y(1-x)[E_{0}^{G}+\Delta E_{1}^{G}-C_{1}^{G}-S+F+\alpha P(Q_{0}-Q_{1})+\alpha P\\ \left(Q_{2}-Q_{0})]+(1-x)(1-y)[E_{0}^{G}-C_{1}^{G}+F+\alpha P(Q_{2}-Q_{0})\right] \end{array}$$

(10) $$\begin{array}{l} E_{G2}=xy(E_{0}^{G}+\Delta E_{0}^{G}-C_{2}^{G})+x(1-y)(E_{0}^{G}+\Delta E_{1}^{G}-C_{2}^{G})\\ +y(1-x)(E_{0}^{G}+\Delta E_{1}^{G}-C_{2}^{G})+(1-x)(1-y)(E_{0}^{G}-C_{2}^{G}) \end{array}$$

(11) $${\bar{E}}_{G}=zE_{G1}+(1-z)E_{G2}$$

In the evolutionary game of Enterprise 1, the expected return $E_{F11}$ of choosing to reduce emissions, the expected return $E_{F12}$ of choosing not to reduce emissions (purchasing carbon indicators), and the average expected return ${\bar{E}}_{F1}$ are expressed as:

(12) $$\begin{array}{l} E_{F11}=yz[E_{0}^{F}+\Delta E_{0}^{F}-C_{1}^{F}+S+P(Q_{0}-Q_{1})]+z(1-y)\\ {}[E_{0}^{F}+\Delta E_{1}^{F}-C_{1}^{F}+S+P(Q_{0}-Q_{1})]+y(1-z)(E_{0}^{F}+\Delta E_{0}^{F}\\ -C_{1}^{F})+(1-y)(1-z)(E_{0}^{F}+\Delta E_{1}^{F}-C_{1}^{F}) \end{array}$$

(13) $$\begin{array}{l} E_{F12}=yz[E_{0}^{F}-C_{2}^{F}-F-P(Q_{2}-Q_{0})]+z(1-y)[E_{0}^{F}\\ -C_{2}^{F}-F-P(Q_{2}-Q_{0})]+y(1-z)(E_{0}^{F}-C_{2}^{F})+(1-y)\\ \left(1-z)(E_{0}^{F}-C_{2}^{F}\right) \end{array}$$

(14) $${\bar{E}}_{F1}=xE_{F11}+(1-x)E_{F12}$$

In the evolutionary game of Enterprise 2, the expected return $E_{F21}$ of choosing to reduce emissions, the expected return $E_{F22}$ of choosing not to reduce emissions (purchasing carbon indicators), and the average expected return ${\bar{E}}_{F2}$ are expressed as:

(15) $$\begin{array}{l} E_{F21}=xz[E_{0}^{F}+\Delta E_{0}^{F}-C_{1}^{F}+S+P(Q_{0}-Q_{1})]+z(1-x)\\ {}[E_{0}^{F}+\Delta E_{1}^{F}-C_{1}^{F}+S+P(Q_{0}-Q_{1})]+x(1-z)(E_{0}^{F}+\Delta E_{0}^{F}\\ -C_{1}^{F})+(1-x)(1-z)(E_{0}^{F}+\Delta E_{1}^{F}-C_{1}^{F}) \end{array}$$

(16) $$\begin{array}{l} E_{F22}=xz[E_{0}^{F}-C_{2}^{F}-F-P(Q_{2}-Q_{0})]+z(1-x)[E_{0}^{F}\\ -C_{2}^{F}-F-P(Q_{2}-Q_{0})]+x(1-z)(E_{0}^{F}-C_{2}^{F})+(1-x)\\ \left(1-z)(E_{0}^{F}-C_{2}^{F}\right) \end{array}$$

(17) $${\bar{E}}_{F2}=yE_{F21}+(1-y)E_{F22}$$

The dynamic evolutionary game among Enterprise 1, Enterprise 2, and the government can be described by means of the replication dynamic equation. The replication dynamic equation of Enterprise 1 is:

(18) $$\begin{array}{ll} F(x) & =dx/dt\\ & =x(1-x)[z(S+F+PQ_{2}-PQ_{1})+y(\Delta E_{0}^{F}-\Delta E_{1}^{F})\\ & \;+C_{2}^{F}+\Delta E_{1}^{F}-C_{1}^{F}] \end{array}$$

The replication dynamic equation of Enterprise 2 is:

(19) $$\begin{array}{ll} F(y) & =dy/dt\\ & =y(1-y)[z(S+F+PQ_{2}-PQ_{1})+x(\Delta E_{0}^{F}-\Delta E_{1}^{F})\\ & \;+C_{2}^{F}+\Delta E_{1}^{F}-C_{1}^{F}] \end{array}$$

The replication dynamic equation of the government is:

(20) $$\begin{array}{ll} F(z) & =dz/dt\\ & =z(1-z)[(1-xy)(F+\alpha PQ_{2})-x(1-y)(S+\alpha PQ_{1})\\ & \;-(1-x-y)\alpha PQ_{0}-y(S+\alpha PQ_{1})+C_{2}^{G}] \end{array}$$

The game between Enterprise 1, Enterprise 2, and the government can be represented by a differential equation composed of F(x), F(y), and F(z). Through the local stability of the Jacobian matrix, the stable strategy of the system evolution is obtained [73], where J is expressed as:

$$J=\left\{\begin{array}{ccc} \begin{array}{l} (1-2x)[z(S+F+PQ_{2}-\\ PQ_{1})+y(\Delta E_{0}^{F}-\Delta E_{1}^{F})+\\ C_{2}^{F}+\Delta E_{1}^{F}-C_{1}^{F}] \end{array} & x(1-x)(\Delta E_{0}^{F}-\Delta E_{1}^{F}) & x(1-x)(S+F+PQ_{2}-PQ_{1})\\ y(1-y)(\Delta E_{0}^{F}-\Delta E_{1}^{F}) & \begin{array}{l} (1-2y)[z(S+F+PQ_{2}\\ -PQ_{1})+x(\Delta E_{0}^{F}-\Delta E_{1}^{F})\\ +C_{2}^{F}+\Delta E_{1}^{F}-C_{1}^{F}] \end{array} & y(1-y)(S+F+PQ_{2}-PQ_{1})\\ \begin{array}{l} z(1-z)[(S+\alpha PQ_{1})(y-1)\\ -y(F+\alpha PQ_{2})+\alpha PQ_{0}] \end{array} & \begin{array}{l} z(1-z)[(S+\alpha PQ_{1})(x-1)\\ -x(F+\alpha PQ_{2})+\alpha PQ_{0}] \end{array} & \begin{array}{l} \left(1-2z)[(1-xy)(F+\alpha PQ_{2}\right)\\ -x(1-y)(S+\alpha PQ_{1})-(1-\\ x-y)\alpha PQ_{0}-y(S+\alpha PQ_{1})\\ +C_{2}^{G}] \end{array} \end{array}\right\}$$

Set the dynamic equations of Enterprise 1, Enterprise 2, and the government to be zero; eight partial equilibrium solutions can be obtained [74], namely $E_{1}(0,0,0)$, $E_{2}(1,0,0)$, $E_{3}(0,1,0)$, $E_{4}(0,0,1)$, $E_{5}(0,1,1)$, $E_{6}(1,0,1)$, $E_{7}(1,1,0)$, and $E_{8}(1,1,1)$. The eigenvalues of the Jacobian matrix obtained from the equilibrium points are shown in Table 7.

When $\Delta E_{1}^{F}+C_{2}^{F}>C_{1}^{F}$ and $C_{2}^{G}>S$, that is, the sum of the income of some enterprises in reducing emissions and the cost of not reducing emissions (purchasing carbon quotas) is higher than the cost of implementing emission reduction, and the cost of the government’s failure to implement regulations on enterprises is higher than the government’s subsidies for emission reduction enterprises, there is an ESS in the Jacobian matrix, namely $E_{8}(1,1,1)$, as shown in Table 8. The corresponding evolution and stabilization strategies of the government, Enterprise 1, and Enterprise 2 are (regulation, emission reduction, and emission reduction).

## 4. Simulation Analysis

In order to analyze the emission reduction cost, government regulation, punishment intensity, subsidy intensity, and stable operation trajectory of carbon trading under carbon allowances for different enterprises, Matlab R2021b is used to simulate and analyze the evolutionary game model between the enterprise–government two parties and the enterprise–enterprise–government tripartite. In the initial state, the enterprise and government are set to choose different strategies with a certain probability. In order to accurately reflect the evolution trajectory of the system, the time step is set to 0.05. In the simulation analysis of the evolutionary game between the enterprise and the government, set $E_{0}^{F}=100$, $E_{0}^{G}=80$, $\Delta E_{0}^{F}=20$, $\Delta E_{1}^{F}=30$, $\Delta E_{1}^{G}=10$, $\Delta E_{0}^{G}=20$, $C_{2}^{F}=40$, $C_{2}^{G}=40$, S=30, P=20, and α=0.1. The evolutionary game situation is discussed in the following situations.

### 4.1. High Emissions Reduction Costs

**Figure 1:**
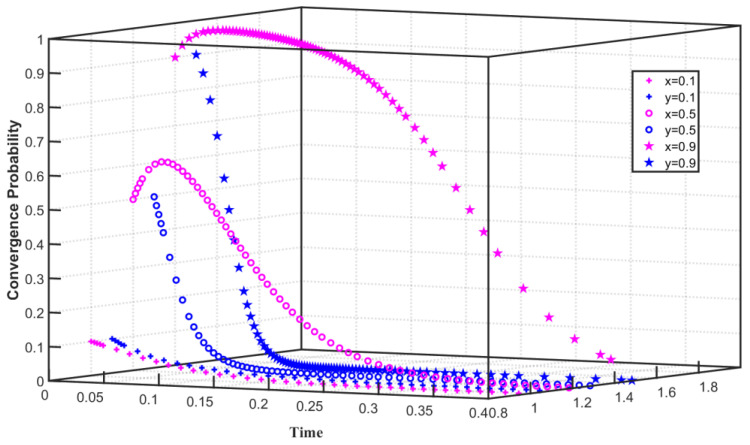
Evolution path of two-party game system under high emission reduction cost and high regulation cost.

Under the constraints of high emission reduction cost and high regulation cost, set $C_{1}^{F}=80$, $C_{1}^{G}=55$, F=40, $Q_{0}=25$, $Q_{1}=24$, and $Q_{2}=26$. As shown in Figure 1, the initial willingness of enterprises to choose carbon emission reduction is x=0.1, the initial willingness of the government to choose the emission reduction regulation for enterprises is y=0.1, x converges to 0 within 0–0.2, and y converges to 0 more slowly. As the initial willingness increases, when x=0.5, x tends to 1 within 0–0.03 but converges to 0 after 0.03. When y=0.5, y converges to 0 within 0–0.1. As the initial willingness is further enhanced when x=0.9, x converges to 1 within 0–0.05 and to 0 after 0.05. When y=0.9, y converges to 0 within 0–0.09. The simulation results show that when the cost of carbon emission reduction is high, the enterprise will eventually converge on not implementing carbon emission reduction. However, as the initial willingness to reduce carbon emission increases, the enterprise initially tends to converge on carbon emission reduction, but when it is affected by the cost of emission reduction, the enterprise finally converges not to carry out carbon emission reduction after 0.05. Affected by the high cost of regulation, the government finally chose not to regulate emission reduction enterprises, but with the increase in the government’s willingness to regulate, the government first chose to regulate and then gradually turned to no regulation.

**Figure 2:**
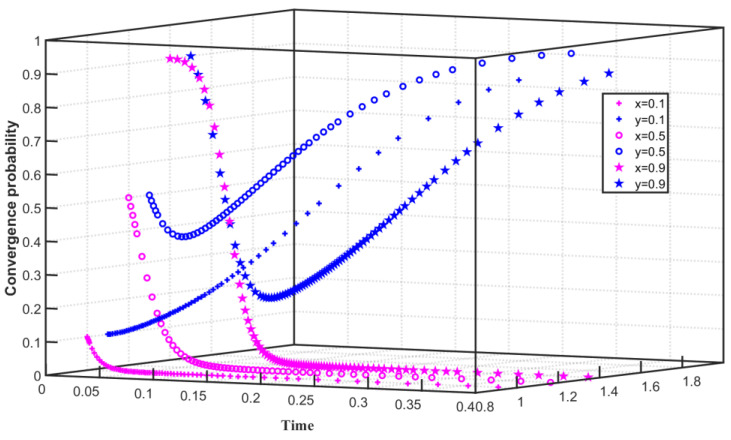
Evolution path of two-party game system under high emission reduction cost and high enterprise punishment.

We set $C_{1}^{F}=180$, F=55, $C_{1}^{G}=55$, $Q_{0}=25$, and $Q_{1}=24$ under the constraints of high emission reduction costs and high enterprise penalties. As shown in Figure 2, the initial willingness of enterprises to choose carbon emission reduction is x=0.1, and the initial willingness of the government to choose to regulate enterprise emission reduction is y=0.1. During 0–0.05, x converges to 0, and y slowly converges to 1. With the improvement of the initial willingness, when x=0.5, x converges to 0 during 0–0.05. When y=0.5, x converges to 0 during 0–0.03 and converges to 1 after 0.03. With the further improvement of the initial willingness, when x=0.9, x decreases slowly in the time of 0–0.01 and quickly converges to 0 after the time of 0.01. When y=0.9, y tends to 0 in 0–0.07 and converges to 1 after 0.07. The simulation results show that when the cost of emission reduction of enterprises is high and the government punishes enterprises that do not reduce emissions, the enterprises tend to choose not to reduce carbon emissions, and the government finally converges on regulating enterprises. However, with the increase in initial willingness, the initial state of the enterprise will change, from initially rapidly converging to no carbon emission reduction to initially preferring carbon emission reduction and then converging to not choosing carbon emission reduction. The initial state of the government will change greatly, from initially tending not to carry out regulation to converging to choose regulation for a longer time.

**Figure 3:**
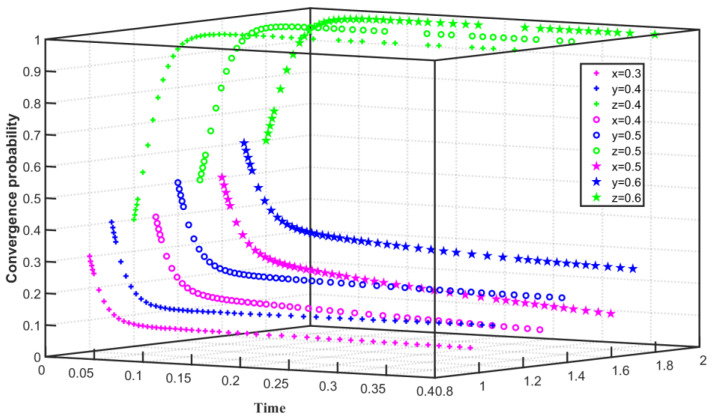
Evolution path of tripartite game system under high emission reduction cost and low regulation cost.

We set $C_{1}^{F}=179$, $C_{1}^{G}=45$, F=40, S=30, $Q_{0}=25$, $Q_{1}=24$, and $Q_{2}=26$ in the assumption that the enterprise’s emission reduction cost is high and the government’s regulatory cost is low. The three-way evolution path of carbon trading is shown in Figure 3. Assuming that the initial willingness of Enterprise 1 to reduce carbon emissions is x=0.3, the initial willingness of Enterprise 2 to reduce carbon emissions is y=0.4, and the initial willingness of the government to regulate is z=0.4, x, and y slowly converge to 0, and z converges to 1 within 0–0.08. With the improvement of the initial willingness of Enterprise 1, Enterprise 2 and the government, that is x=0.5, y=z=0.6, x and y also slowly converge to 0, and z converges to 1 between 0 and 0.07. The simulation results show that if the cost of carbon emission reduction is high, the enterprise will not choose carbon emission reduction but choose to purchase carbon indicators. However, when the enterprise chooses carbon emission reduction willingness to be low, the speed of the enterprise converges on the purchase of carbon indicators is accelerated. When the willingness of enterprises to choose carbon emission reduction increases, the speed of the enterprises converge on the purchase of carbon indicators slows down. However, the choice of the government to regulate the carbon emissions of enterprises is not affected by the level of emission reduction costs of enterprises and is affected by the low cost of government-to-enterprise regulation. With the improvement of the government’s willingness to regulate, the speed of the government’s convergence to regulation is accelerated.

### 4.2. Low Carbon Emission Reduction Costs

**Figure 4:**
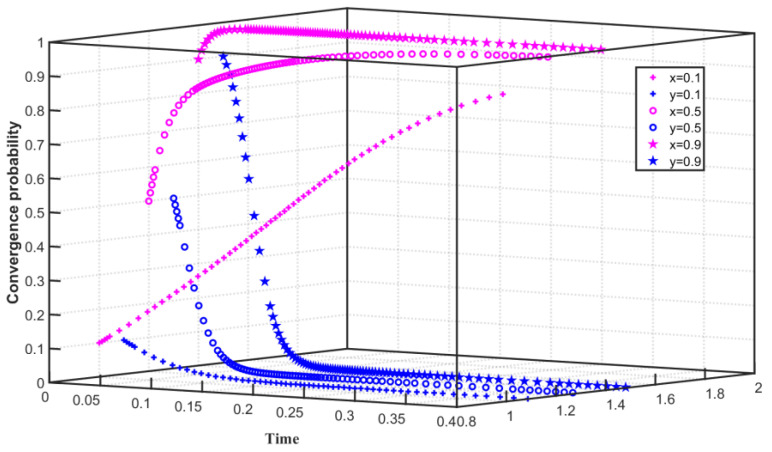
Evolution path of two-party game system under low emission reduction cost and high regulation cost.

Under the constraints of low emission reduction cost and high regulation cost, we set $C_{1}^{F}=55$, $C_{1}^{G}=55$, F=40, $Q_{0}=25$, $Q_{1}=24$, and $Q_{2}=26$. As shown in Figure 4, the initial willingness of enterprises to choose carbon emission reduction is x=0.1, and the initial willingness of the government to choose to regulate enterprise emission reduction is y=0.1. x slowly converges to 1 and y converges to 0 within a short time of 0–0.08. With the improvement of the initial willingness, when x=0.5, x tends to 1 rapidly within 0–0.05 and slowly converges to 1 after 0.05. When y=0.5, y converges to 0 within a short time of 0–0.07. With the further improvement of the initial willingness, when x=0.9, x converges to 1 in a short time of 0–0.05. When y=0.9, y converges to 0 within a short time of 0–0.08. The simulation results show that when the enterprise’s emission reduction cost is low and the government’s regulation cost for emission reduction enterprises is high, the enterprise converges to choose carbon emission reduction, and the government converges to choose not to carry out carbon regulation. With the increase in initial willingness, the time for enterprises to choose not to reduce carbon emissions is prolonged, and the time for governments to converge on carbon emission reduction regulations is shortened.

**Figure 5:**
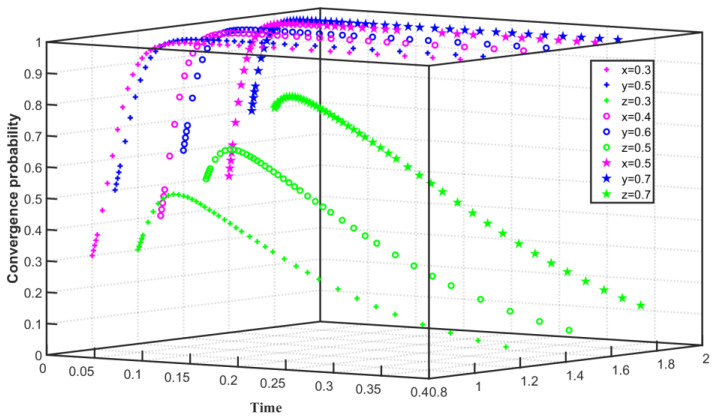
Evolution path of tripartite game system under low emission reduction cost and high government subsidy.

Assuming that the emission reduction cost of the enterprise is low and the government subsidizes the emission reduction enterprise relatively high, we set $C_{1}^{F}=50$, S=50, $C_{1}^{G}=55$, F=40, $Q_{0}=25$, $Q_{1}=24$, and $Q_{2}=26$. The three-party evolution path of carbon trading is shown in Figure 5. The initial willingness of Enterprise 1 to choose carbon emission reduction is x=0.3, the initial willingness of Enterprise 2 to choose carbon emission reduction is y=0.5, and the initial willingness of the government to regulate emission reduction is z=0.3. x converges to 1 in 0–0.08 time, y converges to 1 in 0–0.07 time, z tends to 1 in 0–0.04 time, and gradually converges to 0 after 0.04. With the continuous improvement of the initial willingness of the three parties, when the willingness of x is increased to 0.5, the willingness of y is increased to 0.7, and the willingness of z is increased to 0.7, x converges to 1 within 0–0.04 time, *y* converges to 1 in a shorter time of 0–0.03, and z exhibits a parabolic shape, that is, z tends to 1 in the time of 0–0.01, and slowly tends to 0 after the time of 0.01. Affected by the initial willingness, the inflection point time for z to converge from 1 to 0 is gradually shortened, and the game equilibrium of Enterprise 1, Enterprise 2, and the government finally reaches (emission reduction, emission reduction, non-regulation). The simulation results show that the cost of enterprise emission reduction is lower than the sum of the cost of non-carbon emission reduction and the increase in enterprise emission reduction, and the government’s subsidies to emission reduction enterprises are higher than the sum of the government’s carbon trading revenue and the cost of the government’s non-regulation loss, Enterprise 1 and Enterprise 2 converged on the choice of carbon emission reduction, and the positive actions of the enterprises also affected the government. At first, the government chose to regulate carbon emission reduction for a period of time, but due to the high government subsidies for emission reduction enterprises, the government gradually converged on the choice of not regulating enterprises.

**Figure 6:**
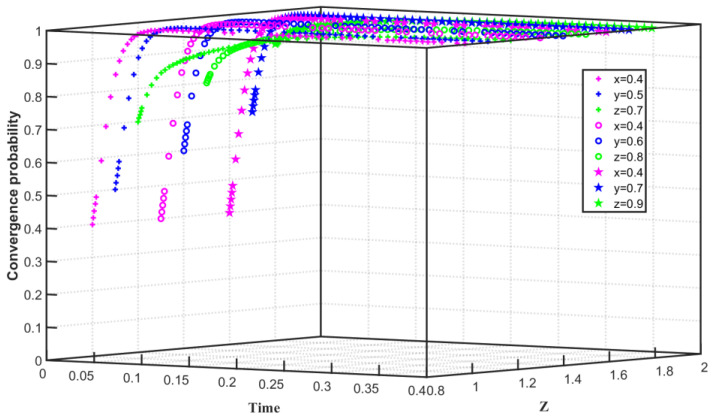
Evolution path of tripartite game system under low emission reduction cost and low regulation cost.

We set $C_{1}^{F}=60$, $C_{1}^{G}=45$, S=30, F=40, $Q_{0}=25$, $Q_{1}=24$, and $Q_{2}=26$ in the assumption that the enterprise’s emission reduction cost is low and the government’s regulatory cost to the enterprise is low. The three-way evolution path of carbon trading is shown in Figure 6. Assuming that the initial willingness of Enterprise 1 to choose carbon emission reduction is x=0.4, the initial willingness of Enterprise 2 to choose carbon emission reduction is y=0.5, and the initial willingness of the government to choose regulation is z=0.7. x converges to 1 in 0–0.08 time, y converges to 1 in 0–0.07 time, z converges to 1 in 0–0.25 time. With the continuous improvement of the initial willingness of Enterprise 2 and the government, when the initial willingness of Enterprise 2 is increased to y=0.6, and the initial willingness of the government is increased to z=0.8, the speed of convergence of x, y, and z to 1 accelerates. With the further improvement of the initial willingness of Enterprise 2 and the government, the initial willingness of Enterprise 2 is increased to y=0.7, and the initial willingness of the government is increased to z=0.9. x converges to 1 in 0–0.06 time, y converges to 1 in 0–0.04 time, z converges to 1 in 0–0.15 time, and the speed of x, y, z convergence to 1 is further accelerated. The simulation results show that when the cost of enterprises implementing emission reduction is lower than the sum of the benefits of some enterprises in reducing emissions and the cost of enterprises not reducing emissions (purchasing carbon quotas), and the cost of the government’s non-implementation of regulations on enterprises is higher than the government’s subsidies for emission reduction enterprises, Enterprise 1, Enterprise 2, and the government eventually converge to 1.That is, the enterprises choose to reduce carbon emissions, and the government chooses to regulate the enterprise. At the same time, with the increase in the initial willingness, the willingness of enterprises to choose carbon emission reduction and the government to choose regulation gradually become stronger. However, affected by the interests of enterprises, the convergence speed of choosing carbon emission reduction is faster than that of the government.

### 4.3. High Free Quota

**Figure 7:**
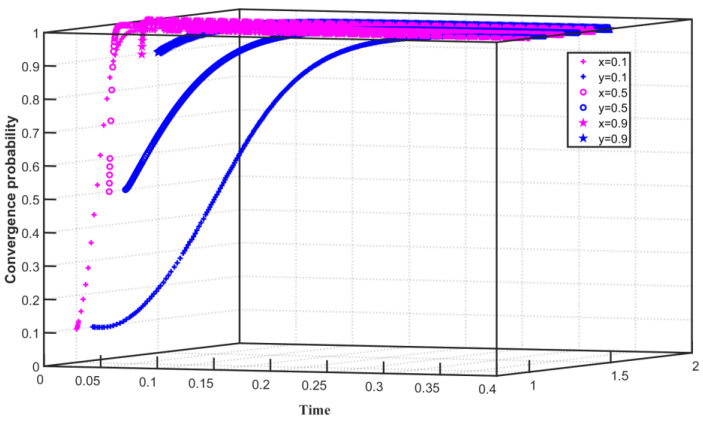
Evolution path of two-party game system under high free quota and low carbon emission reduction.

Under the restriction of high free quota and low carbon emission reduction, we set $Q_{0}=70$, $Q_{1}=15$, $Q_{2}=75$, $C_{1}^{F}=60$, $C_{1}^{G}=55$, and F=40. As shown in Figure 7, the initial willingness of enterprises to choose carbon emission reduction is x=0.1, and the initial willingness of the government to choose to regulate enterprise emission reduction is y=0.1. x converges to 1 in time 0–0.05 and y converges to 1 in time 0–0.3. As the enterprise’s initial willingness to choose carbon emission reduction increases, when x=0.5, x converges to 1 in 0–0.007 time. As the government’s initial willingness to choose carbon regulation increases, when y=0.5, y converges to 1 in 0–0.02 time. With the further improvement of the initial willingness, when x=0.9, x converges to 1 in a short time of 0–0.003. When y=0.9, y converges to 1 within 0–0.01. The simulation results show that when the government’s free carbon quota for enterprises is high and the carbon emission reduction is low, the enterprise tends to choose carbon emission reduction, and the government chooses to regulate enterprise. With the improvement of initial willingness, the convergence speed of enterprises choosing carbon emission reduction is shortened, and the convergence speed of governments choosing carbon emission reduction regulation is also shortened.

## 5. Conclusions

This paper incorporates government behavior as an important factor in carbon trading into a unified analysis framework, innovatively constructs an evolutionary game model between the enterprise–government two parties and the enterprise–enterprise–government tripartite, and explores the multi-agent behavioral strategy path of carbon trading. Simultaneously, a scenario simulation is carried out on the behaviors of enterprises and governments with different costs and different carbon quotas in the game models of enterprise–government and enterprise–enterprise–government. The stable operation path and system evolution trajectory of carbon trading with different intensities of enterprise and government behaviors are intuitively analyzed, which can provide feasible suggestions for enterprise carbon trading environmental policy choices. The main conclusions are as follows:

(1) In the two-party evolutionary game model, the carbon trading behavior is affected by the joint action of the enterprise and the government. Enterprise behavior is closely related to its cost and benefit. The enterprise chooses not to reduce carbon emission when the enterprise’s carbon emission reduction cost is high. The enterprise chooses to reduce carbon emission as the enterprise’s emission reduction cost is low. Government behavior is also constrained by the cost of regulating enterprise emission reductions. When the cost of government regulation is high, the government tends to choose not to regulate enterprises. The revenue of enterprises in carbon trading is relatively large, and the government obtains higher revenue from carbon trading on the condition that the free carbon allowances given by the government to enterprises are high and the carbon emission reduction of enterprises is low. Therefore, the enterprises converge on the choice of carbon emission reduction, and the government converges on the choice to regulate emission reduction enterprises.
(2) In the three-party evolutionary game model, the convergence of the two enterprises and the government to the game equilibrium state ultimately depends on their own costs and benefits, and the carbon trading behavior is still subject to the joint action of the two enterprises and the government. Enterprise behavior is closely related to its cost and benefit. When the emission reduction costs of the two enterprises are high, both enterprises choose not to reduce carbon emissions. When the emission reduction costs of the two enterprises are low, both enterprises choose to reduce carbon emissions. Government behavior is not only related to subsidies to emission reduction enterprises but also affected by the cost of emission reduction regulations for enterprises. When government subsidies to enterprises are high, the government chooses not to regulate enterprises. When the cost of regulation is low, the government tends to choose to regulate enterprises.
(3) The difference in initial willingness mainly affects the speed at which the subject converges to the steady state. When enterprises converge on the choice of carbon emission reduction, with the continuous improvement of the initial willingness, the enterprises will increase the speed of convergence on the stabilization strategy. Correspondingly, when the government converges on choosing to regulate enterprises, with the continuous improvement of the initial willingness, the government will also accelerate the speed of convergence to a steady state. However, when enterprises converge on choosing not to reduce carbon emissions, with the increase in initial willingness, enterprises initially tend to choose carbon emissions reduction and then converge to no carbon emissions reduction, which slows down the speed of convergence to no carbon emissions reduction. When the government chooses not to regulate, the time for the government to converge on not to regulate is prolonged as the government’s willingness to regulate increases.

The following policy implications can be drawn from the above conclusions:

(1) The carbon emission cost of enterprises should be reduced. As a new carbon market model, the healthy operation of carbon trading can drive market entities to actively participate in carbon trading [75], increase the activity of carbon market trading, realize the rationality of carbon allowance pricing, and give full play to the guidance mechanism of carbon price on emission reduction strategies [76]. It forces enterprises to increase R&D expenditures [77], develop emission reduction technologies, reduce the cost of carbon emission reduction, urge enterprises to actively reduce carbon emission, and form a benign interaction in the carbon trading market.
(2) The vitality of enterprises to reduce carbon emissions should be enhanced. At this stage, the industry structure included in the national carbon market is relatively simple, with the power generation industry as the main industry. In the future, more energy-intensive industries will be introduced to enrich the market transaction entities [78], enhance the liquidity of the carbon market, reshape the supply and demand relationship of carbon trading, and increase the trading volume of the carbon market. At the same time, most of the current carbon quota allocation methods are based on free allocation [67], and later consideration will be given to introducing paid allocation mechanism to optimize the rationality of quota allocation and enhance the initiative of enterprises to reduce carbon emissions.
(3) The abilities of regulation and fulfillment for the government should be stimulated. At present, the government has successively issued the “Opinions of the Central Committee of the Communist Party of China and the State Council on Completely, Accurately and Comprehensively Implementing the New Development Concept and Doing a Good Job in Peaking and Carbon Neutralization”, “Interim Measures for the Administration of Carbon Emissions Trading”, and “Administrative Measures for Carbon Emissions Trading (for Trial Implementation)” and other documents [79], in order to ensure the efficient operation of the carbon market, it is still necessary to further improve the regulations at the carbon trading level [80], build a service-oriented government, strengthen the government’s power to supervise carbon trading behavior [81], and optimize public services.

This paper aims to provide a theoretical analysis framework for enterprise carbon trading behavior based on evolutionary game theory. However, limited by real data, the parameter setting in this paper only combines the theoretical analysis of the evolutionary game model and the setting of practical experience so as to analyze the evolution of carbon trading in different situations. The empirical testing of theoretical results combined with real data is the next research direction using this framework.

## Figures and Tables

**Table 1:** Model parameters and definitions.

Parameter	Definition
$E_{0}^{F}$	Comprehensive income when the enterprise does not reduce emissions (purchase carbon quotas)
$\Delta E_{0}^{F}$	Increased comprehensive income when all enterprises choose to reduce emissions at the same time
$\Delta E_{1}^{F}$	$\mathrm{Enterprise}\;\mathrm{income}\;\mathrm{when}\;\mathrm{some}\;\mathrm{enterprises}\;\mathrm{choose}\;\mathrm{not}\;\mathrm{to}\;\mathrm{reduce}\;\mathrm{emissions}\;(\mathrm{purchase}\;\mathrm{carbon}\;\mathrm{quotas})\;\mathrm{and}\;\mathrm{some}\;\mathrm{enterprises}\;\mathrm{reduce}\;\mathrm{emissions}\;(\Delta E_{0}^{F}>\Delta E_{1}^{F}>0)$
$E_{0}^{G}$	Government benefits when enterprises do not reduce emissions (purchase carbon credits)
$\Delta E_{0}^{G}$	$\mathrm{Increased}\;\mathrm{government}\;\mathrm{revenue}\;\mathrm{when}\;\mathrm{all}\;\mathrm{enterprises}\;\mathrm{choose}\;\mathrm{to}\;\mathrm{reduce}\;\mathrm{emissions}\;\mathrm{at}\;\mathrm{the}\;\mathrm{same}\;\mathrm{time}\;(\Delta E_{0}^{G}>0$)
$\Delta E_{1}^{G}$	Government benefits when some enterprises do not reduce emissions (purchase carbon quotas) and when some enterprises choose to reduce emissions
S	Government subsidies for emission reduction enterprises
F	The government’s penalties for enterprises that do not reduce emissions
$C_{1}^{G}$	Regulatory costs for government supervision of enterprise emission reductions
$C_{2}^{G}$	The cost of the government’s failure to implement regulations on whether enterprises reduce emissions
$C_{1}^{F}$	Costs for enterprises to implement emission reduction
$C_{2}^{F}$	The cost of enterprises not reducing emissions (purchasing carbon credits)
$Q_{0}$	Carbon allowances allocated by the government to enterprises for free
$Q_{1}$	Carbon emissions when enterprises actively reduce emissions
$Q_{2}$	Carbon emissions when enterprises do not reduce emissions
P	Carbon trading price

**Table 2:** Evolutionary game payment matrix of carbon trading between government and enterprise.

Strategy		Government	
		Regulation (y)	Non-Regulation (1−y)
Enterprise	Reduction (x)	$E_{0}^{F}+\Delta E_{0}^{F}-C_{1}^{F}+S+P(Q_{0}-Q_{1})$	$E_{0}^{F}+\Delta E_{0}^{F}-C_{1}^{F}$
		$E_{0}^{G}+\Delta E_{0}^{G}-C_{1}^{G}-S+\alpha P(Q_{0}-Q_{1})$	$E_{0}^{G}+\Delta E_{0}^{G}-C_{2}^{G}$
	Purchase (1−x)	$E_{0}^{F}-C_{2}^{F}-F-P(Q_{2}-Q_{0})$	$E_{0}^{F}-C_{2}^{F}$
		$E_{0}^{G}-C_{1}^{G}+F+\alpha P(Q_{2}-Q_{0})$	$E_{0}^{G}-C_{2}^{G}$

**Table 3:** Eigenvalues of the Jacobian matrix of the evolutionary game between the enterprise and the government.

Equilibrium	$\mathrm{Eigenvalues}\;\lambda_{1}$	$\mathrm{Eigenvalues}\;\lambda_{2}$
$E_{1}(0,0)$	$\Delta E_{0}^{F}+C_{2}^{F}-C_{1}^{F}$	$\alpha P(Q_{2}-Q_{0})+F-C_{1}^{G}$
$E_{2}(0,1)$	$\begin{array}{l} \Delta E_{0}^{F}+S+F+P\\ (Q_{2}-Q_{1})+C_{2}^{F}-C_{1}^{F} \end{array}$	$C_{1}^{G}+\alpha P(Q_{0}-Q_{2})-F$
$E_{3}(1,0)$	$C_{1}^{F}-C_{2}^{F}-\Delta E_{0}^{F}$	$\alpha P(Q_{0}-Q_{1})-S-C_{1}^{G}$
$E_{4}(1,1)$	$\begin{array}{l} C_{1}^{F}+P(Q_{1}-Q_{2})-\\ \Delta E_{0}^{F}-C_{2}^{F}-S-F \end{array}$	$C_{1}^{G}+S+\alpha P(Q_{1}-Q_{0})$

**Table 4:** The local stability of the equilibrium point of the evolutionary game between the enterprise and the government.

		*E*_1_(0,0)	*E*_2_(0,1)	*E*_3_(1,0)	*E*_4_(1,1)
Type 1	$\lambda_{1}$	−	+	+	−
	$\lambda_{2}$	−	+	+	−
	Stability	ESS	Saddle point	Saddle point	ESS
Type 2	$\lambda_{1}$	−	−	+	+
	$\lambda_{2}$	+	−	+/−	+/−
	Stability	Unstable point	ESS	Unstable point	Unstable point
Type 3	$\lambda_{1}$	+	+	−	−
	$\lambda_{2}$	+/−	+/−	−	+
	Stability	Unstable point	Unstable point	ESS	Unstable point

**Table 5:** Tripartite evolutionary game payoff matrix with government participation.

Strategy		Enterprise 2	
		Reduction (y)	Purchase (1−y)
Enterprise 1	Reduction (x)	$E_{0}^{G}+\Delta E_{0}^{G}-C_{1}^{G}-S+\alpha P(Q_{0}-Q_{1})$	$\begin{array}{l} E_{0}^{G}+\Delta E_{1}^{G}-C_{1}^{G}-S+F+\\ \alpha P(Q_{0}-Q_{1})+\alpha P(Q_{2}-Q_{0}) \end{array}$
		$E_{0}^{F}+\Delta E_{0}^{F}-C_{1}^{F}+S+P(Q_{0}-Q_{1})$	$E_{0}^{F}+\Delta E_{1}^{F}-C_{1}^{F}+S+P(Q_{0}-Q_{1})$
		$E_{0}^{F}+\Delta E_{0}^{F}-C_{1}^{F}+S+P(Q_{0}-Q_{1})$	$E_{0}^{F}-C_{2}^{F}-F-P(Q_{2}-Q_{0})$
	Purchase (1−x)	$\begin{array}{l} E_{0}^{G}+\Delta E_{1}^{G}-C_{1}^{G}-S+F+\\ \alpha P(Q_{0}-Q_{1})+\alpha P(Q_{2}-Q_{0}) \end{array}$	$E_{0}^{G}-C_{1}^{G}+F+\alpha P(Q_{2}-Q_{0})$
		$E_{0}^{F}-C_{2}^{F}-F-P(Q_{2}-Q_{0})$	$E_{0}^{F}-C_{2}^{F}-F-P(Q_{2}-Q_{0})$
		$E_{0}^{F}+\Delta E_{1}^{F}-C_{1}^{F}+S+P(Q_{0}-Q_{1})$	$E_{0}^{F}-C_{2}^{F}-F-P(Q_{2}-Q_{0})$

**Table 6:** Tripartite evolutionary game payoff matrix without government participation.

Strategy		Enterprise 2	
		Reduction (y)	Purchase (1−y)
Enterprise 1	Reduction (x))	$E_{0}^{G}+\Delta E_{0}^{G}-C_{2}^{G}$	$E_{0}^{G}+\Delta E_{1}^{G}-C_{2}^{G}$
		$E_{0}^{F}+\Delta E_{0}^{F}-C_{1}^{F}$	$E_{0}^{F}+\Delta E_{1}^{F}-C_{1}^{F}$
		$E_{0}^{F}+\Delta E_{0}^{F}-C_{1}^{F}$	$E_{0}^{F}-C_{2}^{F}$
	Purchase (1−x)	$E_{0}^{G}+\Delta E_{1}^{G}-C_{2}^{G}$	$E_{0}^{G}-C_{2}^{G}$
		$E_{0}^{F}-C_{2}^{F}$	$E_{0}^{F}-C_{2}^{F}$
		$E_{0}^{F}+\Delta E_{1}^{F}-C_{1}^{F}$	$E_{0}^{F}-C_{2}^{F}$

**Table 7:** The eigenvalues of Jacobian matrix of tripartite evolutionary game.

Equilibrium	$\mathrm{Eigenvalues}\;\lambda_{1}$	$\mathrm{Eigenvalues}\;\lambda_{2}$	$\mathrm{Eigenvalues}\;\lambda_{3}$
$E_{1}(0,0,0)$	$\Delta E_{1}^{F}+C_{2}^{F}-C_{1}^{F}$	$\Delta E_{1}^{F}+C_{2}^{F}-C_{1}^{F}$	$\begin{array}{l} F+C_{2}^{G}+\alpha P(Q_{2}\\ -Q_{0}) \end{array}$
$E_{2}(1,0,0)$	$C_{1}^{F}-C_{2}^{F}-\Delta E_{1}^{F}$	$\Delta E_{0}^{F}+C_{2}^{F}-C_{1}^{F}$	$\begin{array}{l} F+C_{2}^{G}-S+\alpha P\\ \left(Q_{2}-Q_{1}\right) \end{array}$
$E_{3}(0,1,0)$	$\Delta E_{0}^{F}+C_{2}^{F}-C_{1}^{F}$	$C_{1}^{F}-C_{2}^{F}-\Delta E_{1}^{F}$	$\begin{array}{l} F+C_{2}^{G}-S+\alpha P\\ \left(Q_{2}-Q_{1}\right) \end{array}$
$E_{4}(0,0,1)$	$\begin{array}{l} S+F+C_{2}^{F}+\Delta E_{1}^{F}\\ +P(Q_{2}-Q_{1})-C_{1}^{F} \end{array}$	$\begin{array}{l} S+F+C_{2}^{F}+\Delta E_{1}^{F}\\ +P(Q_{2}-Q_{1})-C_{1}^{F} \end{array}$	$\begin{array}{l} \alpha P(Q_{0}-Q_{2})-C_{2}^{G}\\ -F \end{array}$
$E_{5}(0,1,1)$	$\begin{array}{l} \Delta E_{0}^{F}+C_{2}^{F}+S+F\\ +P(Q_{2}-Q_{1})-C_{1}^{F} \end{array}$	$\begin{array}{l} C_{1}^{F}+P(Q_{1}-Q_{2})-\\ C_{2}^{F}-\Delta E_{1}^{F}-S-F \end{array}$	$\begin{array}{l} S+\alpha P(Q_{1}-Q_{2})\\ -F-C_{2}^{G} \end{array}$
$E_{6}(1,0,1)$	$\begin{array}{l} C_{1}^{F}+P(Q_{1}-Q_{2})-\\ C_{2}^{F}-\Delta E_{1}^{F}-S-F \end{array}$	$\begin{array}{l} C_{2}^{F}+\Delta E_{0}^{F}+S+F\\ +P(Q_{2}-Q_{1})-C_{1}^{F} \end{array}$	$\begin{array}{l} S+\alpha P(Q_{1}-Q_{2})\\ -F-C_{2}^{G} \end{array}$
$E_{7}(1,1,0)$	$C_{1}^{F}-C_{2}^{F}-\Delta E_{0}^{F}$	$C_{1}^{F}-C_{2}^{F}-\Delta E_{0}^{F}$	$\begin{array}{l} \alpha P(Q_{0}-Q_{1})+C_{2}^{G}\\ -S \end{array}$
$E_{8}(1,1,1)$	$\begin{array}{l} C_{1}^{F}+P(Q_{1}-Q_{2})-\\ C_{2}^{F}-\Delta E_{0}^{F}-S-F \end{array}$	$\begin{array}{l} C_{1}^{F}+P(Q_{1}-Q_{2})-\\ C_{2}^{F}-\Delta E_{0}^{F}-S-F \end{array}$	$\begin{array}{l} S+\alpha P(Q_{1}-Q_{0})\\ -C_{2}^{G} \end{array}$

**Table 8:** Local stability of equilibrium points of tripartite evolutionary game.

Eigenvalues	$E_{1}(0,0,0)$	$E_{2}(1,0,0)$	$E_{3}(0,1,0)$	$E_{4}(0,0,1)$	$E_{5}(0,1,1)$	$E_{6}(1,0,1)$	$E_{7}(1,1,0)$	$E_{8}(1,1,1)$
$\lambda_{1}$	+	−	+	+	+	−	−	−
$\lambda_{2}$	+	+	−	+	−	+	−	−
$\lambda_{3}$	+	+	+	−	−	−	+	−
Stability	Saddle point	Unstable point	Unstable point	Unstable point	Unstable point	Unstable point	Unstable point	ESS

## Data Availability

The data are available upon request from the corresponding author.
